# Glucagon receptor signaling regulates weight loss via central KLB receptor complexes

**DOI:** 10.1172/jci.insight.141323

**Published:** 2021-02-22

**Authors:** Shelly R. Nason, Jessica Antipenko, Natalie Presedo, Stephen E. Cunningham, Tanya H. Pierre, Teayoun Kim, Jodi R. Paul, Cassie Holleman, Martin E. Young, Karen L. Gamble, Brian Finan, Richard DiMarchi, Chad S. Hunter, Alexei Kharitonenkov, Kirk M. Habegger

**Affiliations:** 1 Comprehensive Diabetes Center and Division of Endocrinology, Diabetes and Metabolism, Department of Medicine,; 2 Department of Psychiatry and Behavioral Neurobiology, and; 3 Division of Cardiovascular Disease, Department of Medicine, School of Medicine, University of Alabama at Birmingham, Birmingham, Alabama, USA.; 4 Novo Nordisk Research Center Indianapolis, Inc., Indianapolis, Indiana, USA.; 5 Department of Chemistry, College of Arts and Sciences, Indiana University, Bloomington, Indiana, USA.; 6 AK Biotechnologies LLC, Zionsville, Indiana, USA.

**Keywords:** Metabolism, Obesity

## Abstract

Glucagon regulates glucose and lipid metabolism and promotes weight loss. Thus, therapeutics stimulating glucagon receptor (GCGR) signaling are promising for obesity treatment; however, the underlying mechanism(s) have yet to be fully elucidated. We previously identified that hepatic GCGR signaling increases circulating fibroblast growth factor 21 (FGF21), a potent regulator of energy balance. We reported that mice deficient for liver *Fgf21* are partially resistant to GCGR-mediated weight loss, implicating FGF21 as a regulator of glucagon’s weight loss effects. FGF21 signaling requires an obligate coreceptor (β-Klotho, KLB), with expression limited to adipose tissue, liver, pancreas, and brain. We hypothesized that the GCGR-FGF21 system mediates weight loss through a central mechanism. Mice deficient for neuronal *Klb* exhibited a partial reduction in body weight with chronic GCGR agonism (via IUB288) compared with controls, supporting a role for central FGF21 signaling in GCGR-mediated weight loss. Substantiating these results, mice with central KLB inhibition via a pharmacological KLB antagonist, 1153, also displayed partial weight loss. Central KLB, however, is dispensable for GCGR-mediated improvements in plasma cholesterol and liver triglycerides. Together, these data suggest GCGR agonism mediates part of its weight loss properties through central KLB and has implications for future treatments of obesity and metabolic syndrome.

## Introduction

Global obesity rates have tripled since the 1970s (1), a trend that is expected to continue (2). Beyond the accumulation of excess adipose tissue, obesity increases the risks of chronic comorbidities such as type 2 diabetes, cardiovascular disease, and multiple cancers (3). Consistently, weight loss through diet, exercise, surgery, and pharmacological agents has been shown not only to increase quality of life (4–6) but also to positively influence comorbid outcomes (7, 8). Thus, when combined with lifestyle changes, pharmacological agents are a noninvasive tool that can aid in weight loss initiation and management (9, 10).

Initial studies utilizing a single-molecule glucagon receptor (GCGR) and glucagon-like peptide 1 (GLP1) dual agonist have elucidated glucagon as a potent endocrine factor that promotes weight loss (11, 12), making the investigation of downstream pathways that mediate glucagon’s regulation of energy balance highly relevant. Chronic GCGR activation via a selective, long-acting agonist (IUB288) decreases body weight mainly due to a reduction in fat mass (13). Moreover, both acute and chronic GCGR activation (13, 14) increase liver fibroblast growth factor 21 (FGF21) expression (14) and secretion (14, 15), which is partially responsible for the antiobesity properties of GCGR agonism (13).

In the last decade, FGF21 has emerged as an appealing therapeutic for obesity and the metabolic syndrome (16–21) with pleiotropic effects on thermogenesis (22, 23), fatty acid oxidation (21), glucose metabolism (25–27), and body weight (28). FGF21 signals through an FGFR-1c and -3c/β-Klotho (*Klb*) complex (29–32) that is selectively expressed in adipose tissue, liver, pancreas, and brain (33, 34). Early observations focused on peripheral FGF21 action. However, emerging evidence identified central FGF21 signaling increases energy expenditure via sympathetic activity and is required for body weight reductions in diet-induced obese (DIO) mice (35).

Taken together, chronic GCGR signaling potently mediates weight loss, an effect that is mediated in part through liver FGF21. Considering the emerging appreciation for central FGF21 signaling in body weight loss, we hypothesized that GCGR-stimulated FGF21 signals through central KLB to mediate weight loss.

## Results

### Central Klb is dispensable for circadian metabolic phenotype.

**Figure 1:**
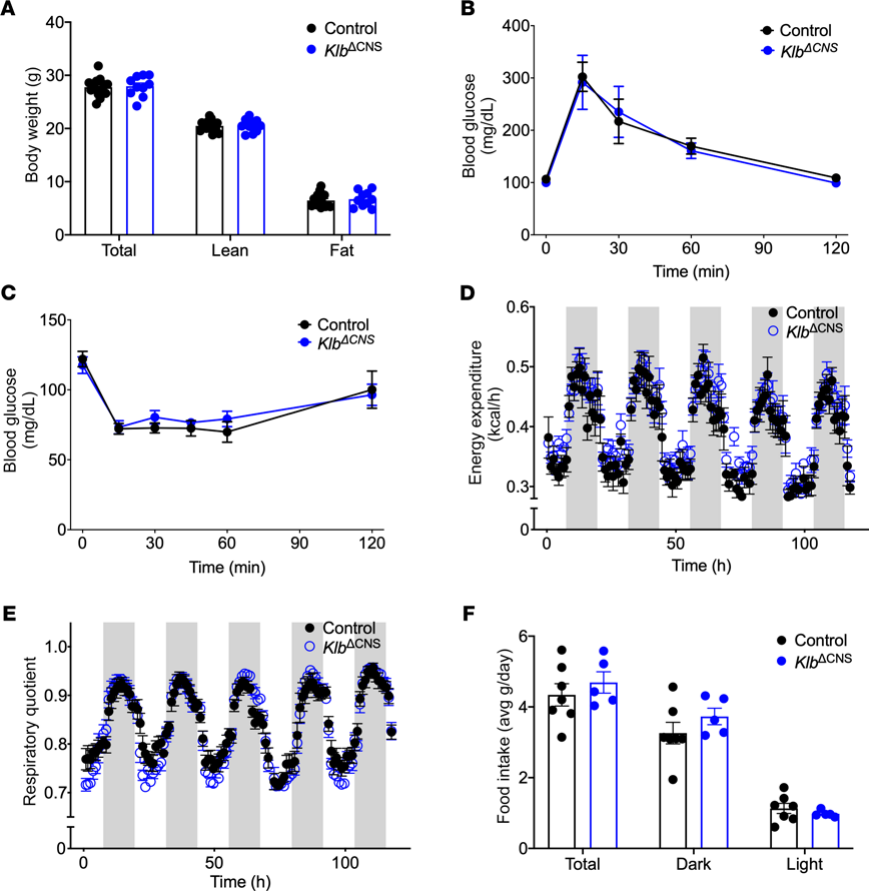
Metabolic profile in *Klb*^ΔCNS^ mice. Body composition (**A**; *n* = 10–14), glucose tolerance (**B**; 5-hour fast, 2 g/kg glucose; *n* = 5–6), and insulin tolerance (**C**; 4-hour fast, 0.5 U/kg insulin; *n* = 5–8) in 8-week-old chow-fed control and *Klb*^ΔCNS^ mice. Energy expenditure (EE) (**D**), respiratory quotient (**E**), and food intake (**F**) in 8-week-old mice (*n* = 5–7). Gray bars depict lights off. All data are represented as mean ± SEM. Two-way ANOVA.

To assess the role of central FGF21 signaling in GCGR-mediated weight loss, we created a mouse model deficient for neuronal *Klb*: C57BL/6J;129/Sv *Klb^tm1^* × B6.Cg-Tg(Syn1-cre)671Jxm/J; *Klb*^ΔCNS^. While exogenous FGF21 reduces body weight via central *Klb* in lean and DIO mice (33, 35), congenital deletion of neuronal *Klb*, and thus loss of central FGF21 signaling, was dispensable in the regulation of body weight, absolute fat, and lean mass in lean, chow-fed mice (Figure 1A). In addition to regulating weight loss, FGF21 is a potent regulator of glucose metabolism (25, 26, 36). However, chow-fed *Klb*^ΔCNS^ mice exhibited similar glucose and insulin tolerance (Figure 1, B and C) as compared to control littermates, suggesting endogenous central FGF21 signaling is also dispensable for glucose homeostasis. Overexpression of FGF21 increases energy expenditure in DIO, but not lean, mice (35). However, consistent with the similar body weights observed between control and *Klb*^ΔCNS^ mice, we found no genotypic alterations in diurnal EE (Figure 1D), respiratory quotient (Figure 1E), or food intake (Figure 1F) between *Klb*^ΔCNS^ and control mice. Together, these data suggest that central *Klb* is dispensable for metabolic homeostasis in lean mice.

**Figure 2:**
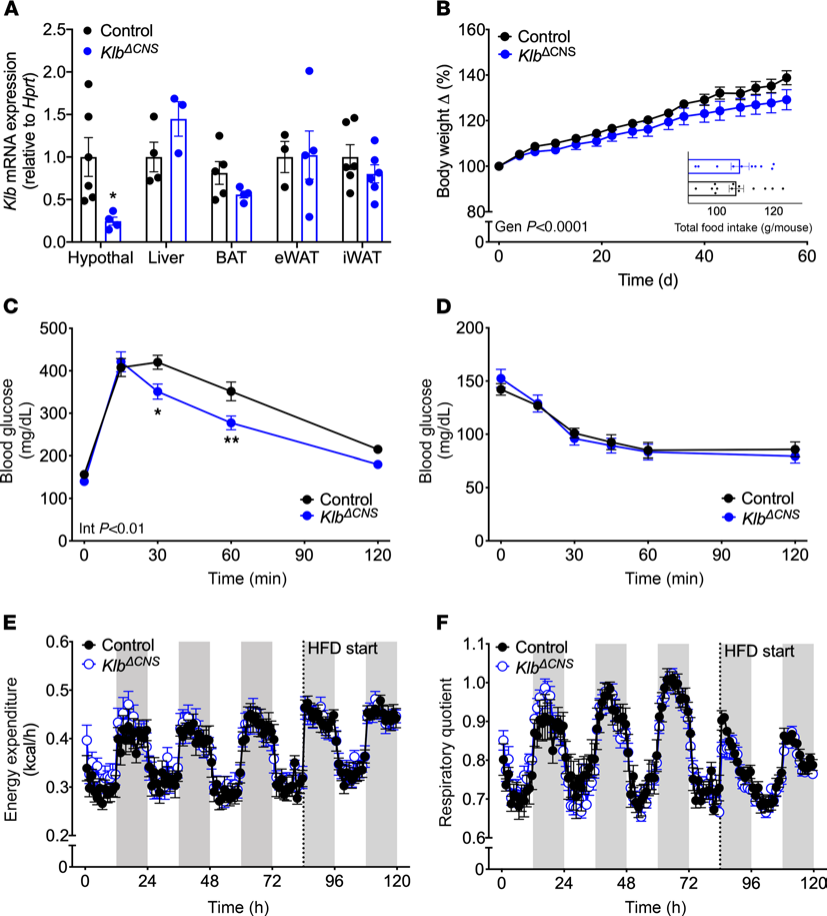
Diet-induced obesity in *Klb*^ΔCNS^ mice. *Klb* expression in hypothalamus and peripheral tissues following 8-week HFD (**A**; *n* = 4–6). Body weight change (% of day 0) (**B**) and food intake (**B**, inset) in HFD-fed mice (*n* = 10–14). Glucose tolerance (**C**; 5-hour fast, 1.5 g/kg glucose) and insulin tolerance (**D**; 4-hour fast, 0.75 U/kg insulin) tests in 16-week-old control and *Klb*^ΔCNS^ DIO mice (*n* = 8–13). EE (**E**) and respiratory quotient (**F**) in 10-week-old control and *Klb*^ΔCNS^ mice (*n* = 5–7). Mice were fed chow diet for first 83 hours. Dotted line indicates start of HFD at 84 hours. Gray bars depict lights off. All data are represented as mean ± SEM. *P < 0.05, **P < 0.01, 2-tailed Student’s *t* test or 2-way ANOVA. (**B**) Main effect of genotype (*P* < 0.0001). (**C**) Interaction of time and genotype (*P* < 0.01). eWAT, epididymal white adipose tissue; iWAT, inguinal white adipose tissue.

*Klb* is expressed by neurons in the suprachiasmatic nucleus (SCN) of the hypothalamus and the hindbrain (33, 37). Supraphysiological levels of FGF21 alter circadian locomotor behavior via central *Klb*, independent of changes in SCN clock gene expression (33). Therefore, we next assessed whether central *Klb* modulates circadian locomotor behavior. Assessment of wheel-running behavior elucidated no genotypic difference in diurnal locomotor pattern or total activity (Supplemental Figure 1, A–B; supplemental material available online with this article; https://doi.org/10.1172/jci.insight.141323DS1). However, we observed a trend toward increased percentage of light phase activity (*P* = 0.06, Supplemental Figure 1C) in *Klb*^ΔCNS^ mice as compared with their littermate controls. Similar periods of endogenous rhythms were observed during constant dark conditions, regardless of genotype (Supplemental Figure 1, D and E). However, there was a significant increase in the alpha length (time from activity onset to activity offset; *P* < 0.05, Supplemental Figure 1F) of *Klb*^ΔCNS^ mice as compared with their littermate controls. Together, this suggests that central *Klb* may regulate components of circadian locomotor behavior, but endogenous FGF21 is not required for general rhythmic homeostasis. A caveat of these studies is the unexpected reduction of *Klb* expression in both the hypothalamus and in peripheral tissues of *Klb*^ΔCNS^ mice compared with control mice (*P* < 0.05, Supplemental Figure 2A, first 2 bars for each tissue). However, unlike lean mice, long-term (≥8 weeks) access to high-fat diet (HFD) selectively reduces *Klb* expression in adipose tissues, while hypothalamic *Klb* expression is maintained (38). Consistently, we observed a significant decrease in *Klb* expression in adipose and liver tissues in control DIO mice (8-week HFD), while hypothalamic *Klb* expression was unchanged (Supplemental Figure 2A, first and third bars). However, there was no further reduction in *Klb* expression between lean and DIO *Klb*^ΔCNS^ mice (Figure 2A and Supplemental Figure 2A, last 2 bars). As such, high-fat feeding induced selective *Klb* reduction in the hypothalamus of DIO *Klb*^ΔCNS^ mice (*P* < 0.05, Figure 2A; and genotype effect *P* < 0.01, Supplemental Figure 2A).

### Central Klb alters diet-induced weight gain.

HFD feeding increases adiposity (diet-induced obesity) and induces metabolic dysregulation. To assess the role of central KLB in the adaptation to this insult, we exposed control and *Klb*^ΔCNS^ mice to an HFD for 8 weeks. Unlike the lean, chow-fed model described above, hypothalamic *Klb* expression was exclusively reduced in these mice as compared with their littermate controls (Figure 2A). Likewise, they were slightly resistant to diet-induced obesity (% weight gain, genotype effect *P* < 0.0001, Figure 2B; and absolute body weight, genotype effect *P* < 0.01, Supplemental Figure 2B) despite similar food intake (Figure 2B, inset) compared to littermate controls. Consistent with their lower body weight, DIO *Klb*^ΔCNS^ mice also displayed improved glucose tolerance (interaction of time and genotype *P* < 0.01, Figure 2C) but similar insulin tolerance as compared to control mice (Figure 2D). We hypothesized that the reduced sensitivity to diet-induced obesity may be due to an increase in EE when *Klb*^ΔCNS^ mice were switched to HFD. Therefore, we conducted indirect calorimetry on lean, chow-fed control and *Klb*^ΔCNS^ mice for 3 days prior to 7 days of high-fat feeding. Similar EE and respiratory energy ratio (RER) were observed between genotypes during the first 3 days (Figure 2, E and F; first 83 hours). When switched to HFD (Figure 2, E and F; 84 hours, dotted line), control mice exhibited an increase in EE and a decrease in respiratory quotient (Figure 2, E and F), as expected. In opposition to our original hypothesis, there were no genotypic differences in EE (Figure 2E, 84–120 hours; and Supplemental Figure 2C), RER (Figure 2F, 84–120 hours; and Supplemental Figure 2D), or diurnal food intake (Supplemental Figure 2E) on HFD. However, we observed a trend for a 58% increase in dark phase locomotor activity (interaction of time of day by genotype, *P* = 0.0563, Supplemental Figure 2F). These data suggest that central *Klb* modulates diet-induced obesity sensitivity but that this regulation is not dependent upon changes in EE.

### Central Klb contributes to GCGR-stimulated weight loss.

Chronic GCGR agonism via IUB288 promotes weight loss and improves lipid homeostasis in DIO mice (13, 14, 39). We previously published that mice deficient for whole-body *Fgf21* (*Fgf21*^–/–^) are refractory to GCGR-mediated prevention of diet-induced obesity (14), and DIO mice deficient for liver *Fgf21* (*Fgf21*^Δliver^) exhibit partial reductions in body weight (13) in response to GCGR agonism, suggesting that FGF21 contributes to the antiobesity effects of GCGR signaling. Based on these findings, we sought to establish the role of central FGF21 signaling, via neuronal *Klb*, in GCGR-stimulated weight loss. Following diet-induced obesity, mice were weight-matched within genotypic groups to receive vehicle or IUB288 treatment for 12 days.

**Figure 3:**
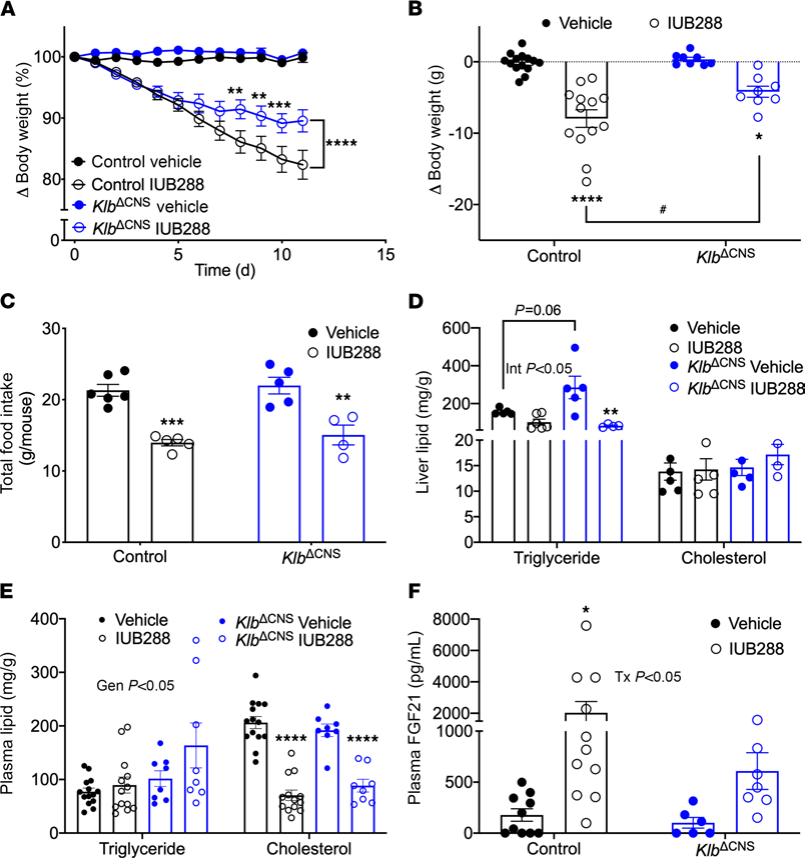
GCGR agonism in *Klb*^ΔCNS^ mice. Body weight change (**A**, %; and **B**, g) and total food intake (**C**) in control and *Klb*^ΔCNS^ DIO mice treated for 12 days with IUB288 (10 nmol/kg/d) (*n* = 8–14). Liver triglycerides (**C**; *n* = 4–6), liver cholesterol (**D**), plasma TG, plasma cholesterol (**E**), and plasma FGF21 (**F**) following 12-day treatment with IUB288. All data are represented as mean ± SEM. *P < 0.05, **P < 0.01, ***P < 0.001, ****P < 0.0001 compared with respective genotypic controls, 2-way ANOVA. ^#^*P* < 0.05 compared with IUB288-treated controls, 2-way ANOVA. (**D**) Interaction of treatment and genotype (*P* < 0.05). (**E**) Main effect of genotype (*P* < 0.05). (**F**) Main effect of treatment (*P* < 0.05). GCGR agonist: IUB288.

Concordant with our previous observations (13, 14, 39), chronic IUB288 administration stimulated robust weight loss in control mice (*P* < 0.0001, % change from day 0, Figure 3A; and body weight change in grams, Figure 3B). In line with our earlier findings in *Fgf21*^Δliver^ mice (13), *Klb*^ΔCNS^ mice displayed a partial reduction in body weight (*P* < 0.0001, Figure 3, A and B; and interaction of time, genotype, and treatment *P* < 0.0001 for absolute body weight loss, Supplemental Figure 2G) as compared with littermate controls, while IUB288 reduced food intake independent of central *Klb* (Figure 3C). This potent weight loss was associated with a decrease in liver triglycerides (TG) (treatment effect *P* < 0.01, Figure 3D, left panel) with no changes in liver cholesterol (Figure 3D, right panel). Interestingly, while *Klb*^ΔCNS^ mice weighed significantly less than littermate controls, they displayed a trend toward increased liver TG (*P* = 0.06), suggesting central KLB may be an important regulator of liver lipid homeostasis. Regardless, liver TG were significantly decreased in IUB288-treated *Klb*^ΔCNS^ mice (*P* < 0.01, Figure 3D, left panel), suggesting central FGF21 signaling is not required for GCGR-mediated reductions in liver TG. Unlike liver lipids, plasma TG were unaltered by IUB288 treatment, regardless of genotype (Figure 3E, left panel). However, we observed an overall increase in plasma TG in DIO *Klb*^ΔCNS^ mice (genotypic effect *P* < 0.05, Figure 3E, left panel). Chronic GCGR agonism also reduced plasma cholesterol, independent of central *Klb* (*P* < 0.0001; Figure 3E, right panel). Taken together, GCGR-mediated improvements in lipid metabolism are independent of central *Klb*.

Central ablation of this receptor may induce compensatory upregulation of the ligand (i.e., FGF21) to act on peripheral tissues. To interrogate potential compensation via increased FGF21, we assessed liver *Fgf21* expression and plasma FGF21 levels in vehicle- and IUB288-treated mice. As expected, IUB288 treatment increased *Fgf21* expression (treatment effect *P* < 0.05, Supplemental Figure 2H) and plasma FGF21 levels (treatment effect *P* < 0.05, Figure 3F) in control mice. Likewise, we observed similar expression (Supplemental Figure 2H) and plasma protein levels in *Klb*^ΔCNS^ mice (Figure 3F), suggesting there is no compensatory upregulation of FGF21 in response to loss of central *Klb*. While plasma FGF21 levels trended lower in *Klb*^ΔCNS^ mice, we were unable to detect a significant difference (*P* = 0.1758) from control IUB288-treated mice (Figure 3F). Together, these data suggest that central *Klb* mediates antiobesity, but not lipid-lowering, properties of GCGR agonism.

### Central KLB antagonism mitigates GCGR-mediated weight loss.

To exclude potential artifacts of developmental *Klb* deficiency, we next employed central (intracerebroventricular, ICV) administration of the competitive pharmacological KLB antagonist, 1153 (40). Due to potential spillover of cerebrospinal fluid into the periphery from ICV delivery, we sought a dose of 1153 that would be subthreshold for peripheral action. Acute FGF21 action improves glucose and insulin tolerance via peripheral (adipose) KLB (41–43). As such, we used glucose tolerance as a readout of peripheral FGF21 action to assess the physiological effects of 1153. Acute subcutaneous pretreatment of FGF21 1 hour (–60 minutes) prior to a glucose challenge (2 g/kg) improved glucose tolerance (44) (AUC *P* < 0.01, Supplemental Figure 3, A and B). Subcutaneous administration of 1153 ten minutes prior to FGF21 pretreatment (–70 minutes) blocked the beneficial effects of FGF21 on glucose tolerance at 3 mg/kg (AUC *P* = 0.99) but not at 0.3 mg/kg (AUC *P* < 0.01) (Supplemental Figure 3, A and B). Therefore, we chose the subthreshold dose of 0.3 mg/kg 1153, as any potential 1153 diffusion to the periphery at this dose should be insufficient to block peripheral FGF21 action.

**Figure 4:**
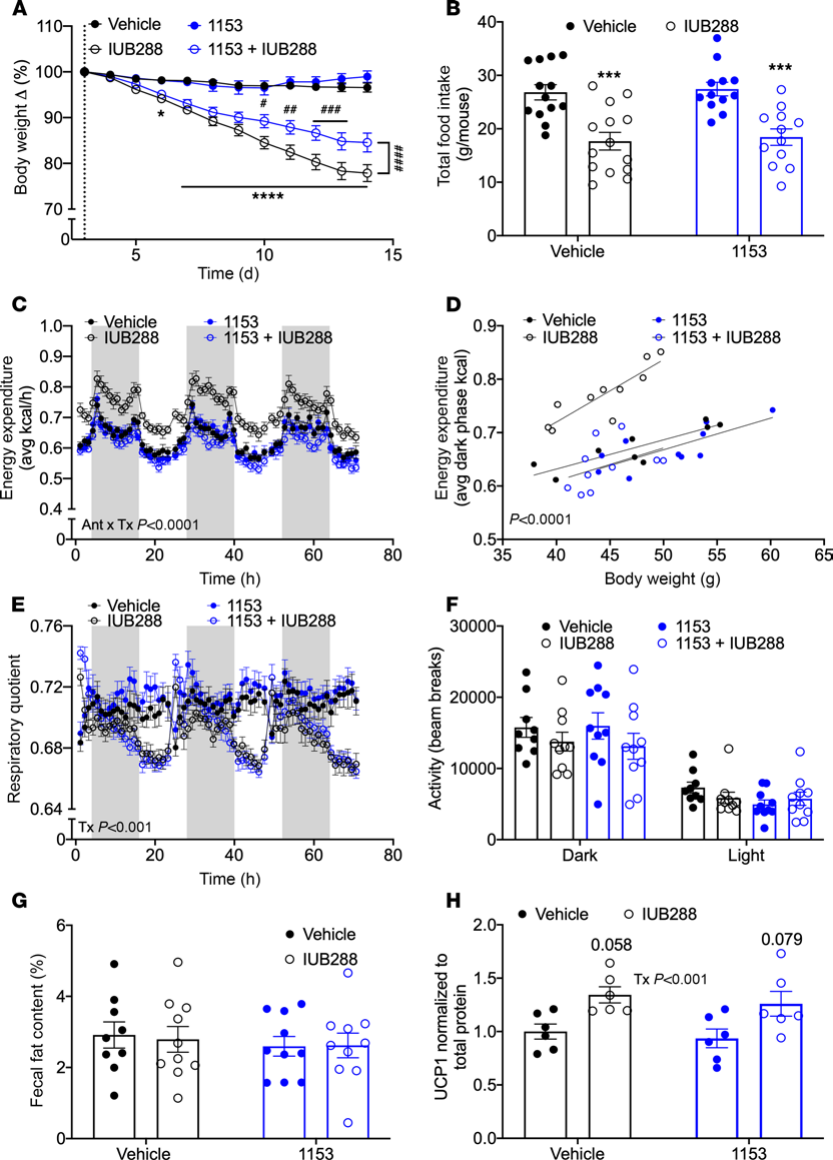
GCGR agonism in mice with KLB antagonism. Body weight change (% of 0, start of IUB288 treatment indicated by dotted line) (**A**, *n* = 12–14) and food intake (**B**) in control and 1153 DIO mice with 14-day minipump ICV 1153 (0.0171 mg/d, equivalent to 0.3 mg/kg) or vehicle administration and treated for 12 days with IUB288 (10 nmol/kg/d). EE (**C** and **D**), respiratory quotient (RQ) (**E**), activity (**F**), and fecal fat content (**G**, %) in mice placed in indirect calorimetry units; measurements taken during days 5–7 of IUB288 treatment. Gray bars depict lights off. BAT UCP1 protein levels normalized to total protein (**H**). All data are represented as mean ± SEM. *P < 0.05, ***P < 0.001, ****P < 0.0001 compared with respective genotypic controls, 2-way ANOVA. ^#^*P* < 0.05, ^##^*P* < 0.01, ^###^*P* < 0.001, ^####^*P* < 0.0001 between IUB288 and 1153 + IUB288 groups, 2-way ANOVA. (**C**) Interaction of treatment and antagonism (*P* < 0.0001). (**D**) Effect of elevation (*P* < 0.0001). (**E**) Main effect of treatment (*P* < 0.001). (**H**) Effect of treatment (*P* < 0.01). GCGR agonist: IUB288. KLB antagonist: 1153.

DIO C57BL6/J mice underwent placement of ICV cannulae and subcutaneous osmotic minipumps. All pumps delivered vehicle or 1153 (0.0171 mg/d, equivalent to 0.3 mg/kg/d) for 14 days. Mice received 1153 for 2 days before the start of IUB288 (day 1; dotted line), to ensure adequate time for KLB antagonism. During the subsequent 12 days, mice received a daily subcutaneous injection of 10 nmol/kg IUB288 or vehicle. As expected, the cannula/pump placement resulted in a slight body weight decrease in all groups; however, we observed no 1153-associated differences (Supplemental Figure 3C). Congruent with our previous findings (13, 14, 39), IUB288 stimulated a significant decrease in body weight in control mice (*P* < 0.0001, day 12 compared with day 1 IUB288 start, Figure 4A). Mice receiving the combination of central 1153 and subcutaneous IUB288 also lost a significant amount of body weight in comparison with their respective control; however, this weight loss was significantly less than control IUB288-treated mice (*P* < 0.0001, Figure 4A). Consistent with our previous findings, chronic GCGR agonism decreased food intake (*P* < 0.001, Figure 4B). However, IUB288-mediated suppression of food intake was maintained in 1153-treated mice, suggesting that IUB288 mediates food intake independent of central FGF21 signaling. Moreover, these data suggest that the partial reductions in body weight are not mediated via differences in food intake.

### Central KLB antagonism mitigates GCGR-mediated EE.

Chronic GCGR agonism increases EE in lean and DIO mice (13, 14). Mice deficient for whole-body FGF21 (*Fgf21*^–/–^) are resistant to IUB288-stimulated EE (14), suggesting the partial reduction in body weight may be due to differences in EE. In the present study, mice treated with IUB288 exhibited body weight–independent increases in EE (interaction of antagonism and IUB288 treatment *P* < 0.0001, Figure 4, C and D), decreased RER (IUB288 treatment effect *P* < 0.001, Figure 4E), similar diurnal activity (Figure 4F), and similar fecal fat content (%, Figure 4G). KLB antagonism alone did not alter these parameters; however, mice with KLB antagonism were resistant to IUB288-stimulated EE (Figure 4, C and D) but not IUB288-mediated reduction in RER (Figure 4E). Moreover, central FGF21 signaling upregulates brown adipose tissue (BAT) uncoupling protein 1 (*Ucp1*) expression (35). There was a modest, but significant, increase in BAT UCP1 in IUB288-treated mice (treatment effect *P* < 0.01, Figure 4H and Supplemental Figure 3E), independent of 1153. This suggests that central FGF21 signaling mediates GCGR-stimulated weight loss via EE but independent of BAT UCP1.

### Central KLB is dispensable for GCGR-mediated improvements in lipid metabolism.

**Figure 5:**
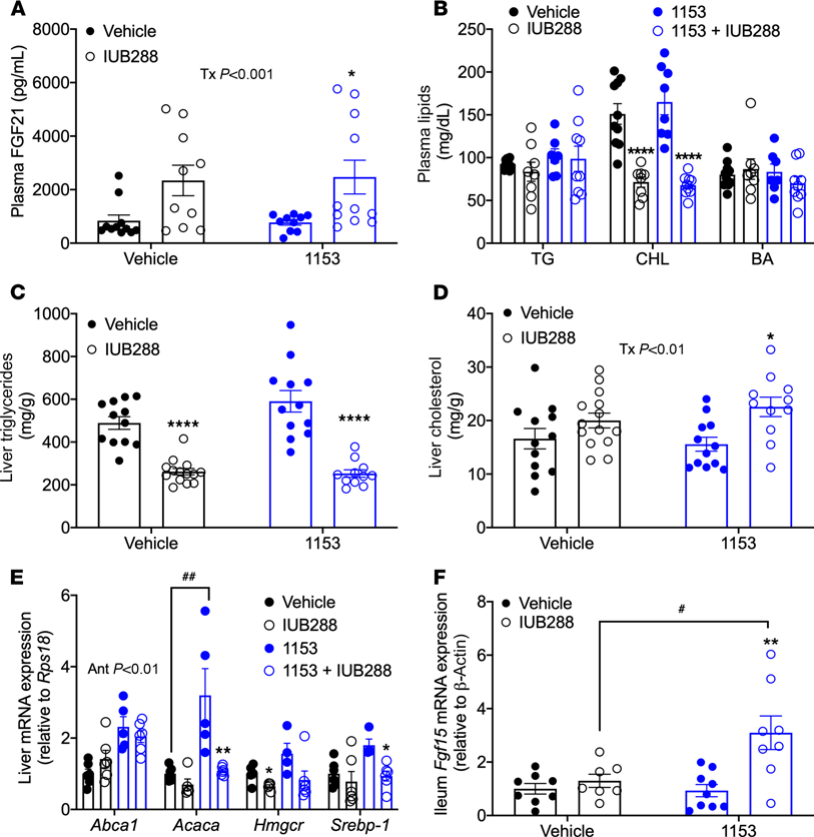
FGF21, lipid, and *Fgf15* profile in mice with KLB antagonism. Plasma FGF21 (**A**), plasma lipids (**B**), liver TG (**C**), liver cholesterol (**D**), liver cholesterol synthesis genes (**E**), and ileum *Fgf15* expression (**F**) in control and 1153 DIO mice with 14-day minipump ICV 1153 (0.0171 mg/d) or vehicle administration and treated for 12 days with IUB288 (10 nmol/kg/d). All data are represented as mean ± SEM. *P < 0.05, **P < 0.01, ****P < 0.0001 compared with respective genotypic controls. ^#^*P* < 0.05, ^##^*P* < 0.01 between vehicle and 1153 groups, 2-way ANOVA. (**A**) Main effect of treatment (*P* < 0.001). (**D**) Main effect of treatment (*P* < 0.01). (**E**) Main effect of 1153 on *Abca1* (*P* < 0.01). GCGR agonist: IUB288. KLB antagonist: 1153. CHL, cholesterol; BA, bile acids.

Consistent with results in *Klb*^ΔCNS^ mice, IUB288 increased plasma FGF21 independent of 1153 (treatment effect *P* < 0.001, Figure 5A), suggesting this regulation is independent of central KLB antagonism. Chronic GCGR agonism is a potent regulator of lipid metabolism, including reducing plasma cholesterol and liver TG (13, 14, 39). We observed a significant and KLB-independent reduction of plasma cholesterol with IUB288 treatment (*P* < 0.0001, Figure 5B, middle panel) and unaltered plasma TG (Figure 5B, left panel). While we had previously observed IUB288-stimulated increases in plasma bile acids (13), this observation did not persist in the current study (Figure 5B, right panel). In the context of liver lipids, IUB288 decreased liver TG regardless of 1153 (*P* < 0.0001, Figure 5C), suggesting GCGR agonism regulates liver TG independent of a central FGF21 signal. Alternatively, IUB288 treatment stimulated a small but significant increase in liver cholesterol (treatment effect, *P* < 0.01, Figure 5D). Expression of genes involved in cholesterol biosynthesis (*Hmgcr* and *Srebp-1*) are decreased with IUB288, regardless of 1153 (Figure 5E), suggesting the increased liver cholesterol is independent of cholesterol biosynthesis.

Liver FGF21 and farnesoid X receptor (FXR) are both downstream pathways that mediate GCGR-stimulated weight loss (13). While we did not observe IUB288-dependent regulation of *Fxr* gene expression, central KLB antagonism increased *Fxr* mRNA (*P* < 0.01, Supplemental Figure 3F). However, despite this increase in gene expression, we observed no 1153 effects on the FXR target genes *Cyp7a1* and *Shp* (Supplemental Figure 3F). Additionally, FGF15/19 also requires the KLB coreceptor complex for signaling (45). While endogenous FGF15/19 is known to signal mainly to the liver, pharmacological levels of FGF19 have also been shown to stimulate EE and weight loss with intravenous (46) and ICV administration (47). In our model, *Fgf15* expression was unperturbed by chronic GCGR agonism or KLB antagonism (Figure 5F). However, *Fgf15* was significantly increased in 1153 mice cotreated with IUB288. These data suggest that FGF15 does not likely play a role in GCGR-mediated weight loss, and *Fgf15* expression in IUB288-treated 1153 mice is likely a result of compensatory upregulation. Together, these data suggest GCGR agonism mediates weight loss, but not improvements in lipid metabolism, via central FGF21 signaling.

### Central KLB is dispensable in GCGR-mediated glucose homeostasis.

**Figure 6:**
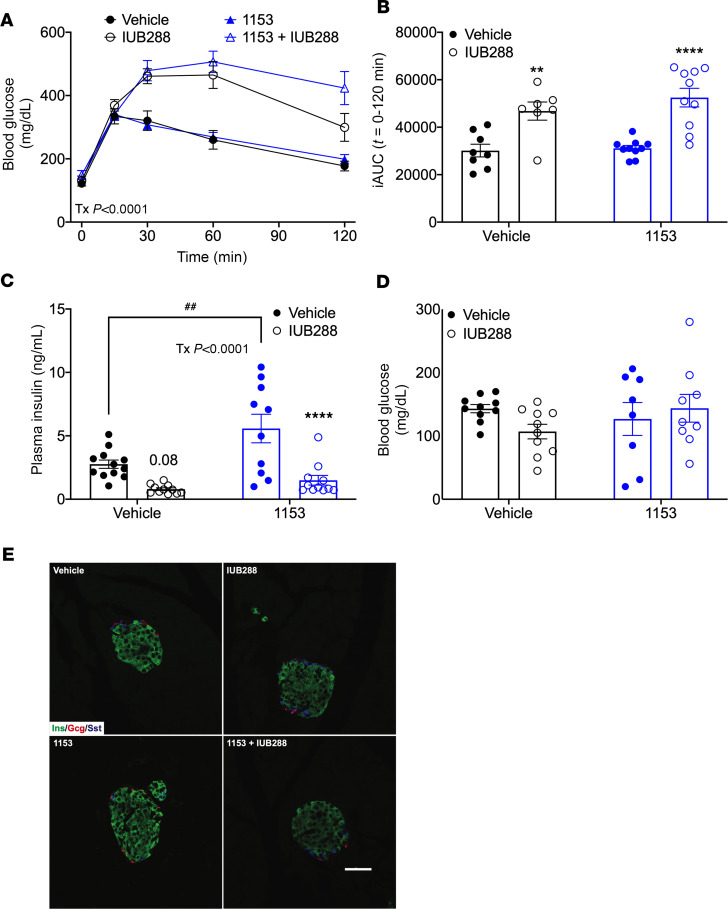
Glucose homeostasis in mice with KLB antagonism. Glucose tolerance test (**A**; 5-hour fast, 1.5 g/kg glucose) and AUC (*t* = 0–120 min) (**B**) in control and 1153 DIO mice with 14-day minipump ICV 1153 (0.0171 mg/d) or vehicle administration and treated for 12 days with IUB288 (10 nmol/kg/d). Plasma insulin (**C**), blood glucose (**D**), and islet fluorescent immunohistochemistry (**E**) following a 2-hour fast. **P < 0.01, ****P < 0.0001 compared with respective genotypic controls. ^##^*P* < 0.01 between vehicle and 1153 groups. (**A**) Main effect of treatment (*P* < 0.0001). GCGR agonist: IUB288. KLB antagonist: 1153. Ins, insulin (green); Gcg, glucagon (red); Sst, somatostatin (blue). Scale bar: 50 μm.

We previously reported that chronic GCGR agonism impairs glucose tolerance (14). In the present study, chronic IUB288-treated mice were also glucose intolerant, independent of KLB antagonism (Figure 6A, effect of treatment *P* < 0.0001; and Figure 6B AUC *t* = 0–120 minutes, *P* < 0.01). Historically, glucagon has been viewed as the main counterregulatory hormone to insulin. However, emerging evidence suggests a more complex relationship of glucagon in glucose homeostasis. Surprisingly, acute glucagon and IUB288 increase insulin secretion (48, 49). Additionally, we have reported that acute and chronic IUB288 improves insulin sensitivity in both lean and DIO mice (28, 35). Consistent with increased insulin sensitivity, chronic GCGR agonism in DIO mice significantly reduced plasma insulin independent of KLB antagonism (treatment effect *P* < 0.0001, Figure 6C). However, 1153 alone increased plasma insulin levels (*P* < 0.01, Figure 6C), despite similar blood glucose levels (Figure 6D) and islet architecture (Figure 6E). Together, these data suggest central KLB regulates circulating insulin levels but is dispensable in GCGR-mediated glucose homeostasis.

## Discussion

Emerging evidence has highlighted the beneficial effects of GCGR signaling on energy balance and lipid metabolism (12, 50), bringing renewed attention to the therapeutic manipulation of the glucagon signaling pathway. Despite these beneficial effects, GCGR monoagonism induces hyperglycemia, which diminishes utility. Therefore, it is increasingly important to understand the downstream mechanisms by which GCGR signaling regulates these metabolic benefits. We previously identified FGF21 as a downstream target of hepatic GCGR signaling and a partial mediator of GCGR-mediated weight loss (13). Liver-derived FGF21 acts centrally to mediate energy expenditure and weight loss (33, 35); thus, we hypothesized that GCGR-mediated FGF21 similarly acts in the brain to regulate this effect.

### Central Klb regulation of energy balance and circadian homeostasis in lean mice.

Since the discovery of FGF21 as a novel endocrine fibroblast growth factor (20), much attention has been given to its physiological role in energy balance and the tissues critical for FGF21 action. FGF21 signaling, via KLB, in adipose tissue is necessary for the beneficial effects of FGF21 on glucose metabolism (42, 51). FGF21 action in the brain regulates both EE (35) and circadian rhythms (33). Studies herein uncovered that conditional developmental deletion of neuronal *Klb* does not alter body weight, glucose homeostasis, EE, or food intake in lean mice. Together these findings suggest that endogenous FGF21 is dispensable in the regulation of unchallenged energy balance or glucose homeostasis.

FGF21 exhibits a diurnal rhythm in (52, 53) and humans (54), and overexpression of FGF21 disrupts circadian locomotor behavior via hypothalamic *Klb* (33). Bookout et al. (33) showed that overexpression of FGF21 reduces total running wheel behavior, with an increase in light phase activity, both of which are normalized in mice deficient for central *Klb*. In the present study, control and *Klb*^ΔCNS^ mice displayed relatively similar diurnal locomotor activity; however, there was a trend toward an increase in light activity (absolute and percentage of total activity) in the *Klb*^ΔCNS^ mice. Additionally, in free-running conditions (constant darkness), *Klb*^ΔCNS^ mice displayed a significant increase in alpha length (less consolidated activity, *P* < 0.05). Although supraphysiological FGF21 levels clearly alter circadian rhythms via central *Klb* (33), data herein suggest that loss of endogenous FGF21 signaling in the brain mediates subtle aspects of circadian rhythms but is not required for general circadian homeostasis. However, these interpretations must be tempered by the suppression of *Klb* expression in non-neuronal tissues.

### Central KLB in diet-induced obesity and GCGR-mediated weight loss.

Mice deficient for whole-body *Klb* (*Klb*^–/–^) (51) or forebrain *Klb* (Camk2a-cre; *Klb*^Camk2a^) (35) are refractory to FGF21-stimulated weight loss. Despite pharmacological FGF21 mediating body weight and EE, mice deficient for physiological FGF21 signaling (*Klb*^–/–^) display similar body weight, EE, and food intake compared with control mice in an unchallenged, chow-fed state (51). Forebrain *Klb*-deficient mice also exhibit no differences in body weight on a chow diet (35), consistent with our results. Although most models display similar diet-induced obesity between control and *Klb*-deficient mice (35, 41, 51), Somm et al. (55) showed that, similar to our *Klb*^ΔCNS^ mice, *Klb*^–/–^ mice are somewhat protected from diet-induced obesity. It should be noted that despite using the same floxed allele, Owen et al. (35) observed no differences in diet-induced obesity between control and *Klb*^Camk2a^ mice. These observed differences may arise from differences in central cre drivers and deserve further investigation. Our observed resistance may be a result of nutrient malabsorption on HFD and increased activity, as we observed no overt differences in food intake or EE and a trend (*P* = 0.0563) for increased (58%) dark phase locomotor activity. Mice deficient for whole-body *Fgf21* (*Fgf21*^–/–^) are more sensitive to diet-induced obesity (56). Thus, we expected a similar phenotype in mice lacking central FGF21 signaling (*Klb*^ΔCNS^ mice). Our observed differences may result from compensatory metabolic adaptations with congenital deletion of *Klb*. In the context of GCGR-mediated weight loss, *Klb*^ΔCNS^ mice exhibited a partial reduction in body weight with chronic IUB288 treatment, suggesting FGF21 is mediating the antiobesity properties of GCGR agonism via a central mechanism. It must be noted that due to their relative diet-induced obesity resistance, *Klb*^ΔCNS^ mice start treatment at a lower body weight; therefore, we cannot exclude *Klb*^ΔCNS^ defending their already reduced body weight as a result of a reduction at baseline. Further, while *Klb* expression was reduced in central and peripheral tissues in lean mice, *Klb* expression was selectively reduced in the hypothalamus in DIO mice, resulting from reduced peripheral *Klb* expression in control DIO mice, and did not alter interpretation of GCGR-mediated weight loss. Additionally, while plasma FGF21 levels were not significantly different in IUB288-treated *Klb*^ΔCNS^ mice compared with control-treated mice (*P* = 0.1758), the altered levels following GCGR-mediated weight loss warrant speculation of potential compensatory metabolic effects in the *Klb*^ΔCNS^ model.

To address this concern, we utilized ICV delivery of a pharmacological inhibitor of KLB, 1153, to mimic our congenital neuronal *Klb* knockout. Consistent with our *Klb*^ΔCNS^ model, ICV administration of 1153 blunted the weight loss and abrogated the EE effects of IUB288, confirming the role of central KLB in GCGR-stimulated weight loss via regulating EE. Importantly, unlike the artifacts observed in the *Klb*^ΔCNS^ model, initial body weight and plasma FGF21 levels were consistent across treatment groups. Although FGF21 upregulates UCP1 via a central mechanism (35), the blunted weight loss observed was independent of BAT UCP1. These data are consistent with emerging literature showing UCP1 is dispensable for FGF21-mediated weight loss (57, 58).

In the present study, we assessed deletion or antagonism of KLB throughout the CNS. As such, it is important to identify which area(s) within the CNS are responsible for mediating these effects. Emerging evidence suggests that *Klb* is expressed in multiple nuclei in the hypothalamus, including the paraventricular nucleus (59), ventromedial hypothalamus (VMH) (59), arcuate nucleus (59), and SCN (33, 37, 59). A recent study identified KLB in the VMH to be necessary for FGF21-mediated suppression of carbohydrate intake but not weight loss. Future studies are required to selectively target KLB complexes in the other hypothalamic nuclei to identify their potential contribution to FGF21-mediated weight loss.

It must be noted that FGF15/19 also signals via KLB (60, 61). Human FGF19 increases EE (46), increases glucose uptake (62), decreases food intake (47), and decreases body weight (47) in rodent models. With 52% homology to FGF19, FGF15 has been shown to decrease cholesterol 7 alpha-hydroxylase expression in the liver, similar to FGF19. However, in opposition to FGF19, FGF15 does not increase glucose uptake in adipocytes (62). With diverging physiological effects, it is unclear if FGF15 modulates body weight loss similar to FGF19. Regardless, ileum *Fgf15* expression was unperturbed with chronic GCGR agonism. While we cannot specifically exclude any potential role of FGF15 in GCGR-mediated weight loss, our data suggest it is unlikely.

### Central KLB in GCGR-mediated improvements in lipid metabolism.

FGF21 and glucagon both beneficially regulate lipid metabolism, such as increasing ketogenesis while decreasing liver TG and plasma cholesterol (13, 39, 63–65). Studies showing FGF21-mediated improvements in lipid metabolism have utilized supraphysiological (35) or pharmacological (63) doses of FGF21. Alternatively, mice deficient for FGF21 show modest (66) or no (67) differences in fasting liver fatty acid oxidation genes or ketogenesis compared with control mice (65), suggesting the physiological actions of FGF21 are distinct from those stimulated by FGF21 at pharmacological levels (65). Consistent with pharmacological actions of GCGR agonism, we observed decreased plasma cholesterol and liver TG following IUB288 treatment. Despite regulating similar lipid endpoints, central KLB is not required for GCGR-mediated reductions in plasma cholesterol and liver TG, as mice with genotypic knockout or pharmacological antagonism of central KLB also exhibit reductions in these lipid parameters with IUB288 treatment. Alternatively, improvements in lipid metabolism may be dependent on body weight loss associated with FGF21, as mice deficient for central *Klb* are refractory to both FGF21-stimulated weight loss and the improvements in lipid metabolism (35). Last, our approach utilizes pharmacological GCGR agonism to produce substantial decreases in plasma cholesterol and liver TG that may overshadow more subtle effects (i.e., central KLB regulation).

### GCGR agonism and KLB antagonism in glucose homeostasis.

While the main endpoint for this study was weight loss, we observed interesting effects of GCGR agonism and central KLB antagonism on parameters in glucose homeostasis. Historically, glucagon has been seen as the main counterregulatory hormone to insulin. Chronic GCGR agonism induces glucose intolerance, fitting this classical view; however, the role of glucagon in glucose metabolism is expanding. We have previously identified that acute and chronic GCGR agonism increases insulin sensitivity (48). Additionally, we and others have identified that acute glucagon or GCGR agonism increases insulin secretion (48, 49), mediated via pancreatic GCGR and GLP1 receptor (49). In the present study, we found that chronic GCGR agonism in DIO mice decreased plasma insulin, which may be a result of weight loss. Clinical utility of GCGR agonism is limited by its negative role in glucose metabolism. As such, future studies are needed to interrogate the role of chronic GCGR agonism in reducing insulin levels.

Additionally, FGF21 improves glucose tolerance and insulin sensitivity (41, 42, 68). While central KLB antagonism was not necessary for GCGR-mediated reductions in plasma insulin, central 1153 alone increased circulating insulin, independent of blood glucose levels. This suggests endogenous FGF21 may regulate insulin levels via a brain/pancreas axis. Future studies are warranted to dissect the contribution of direct (sympathetic) versus indirect (hormone) regulation of the FGF21 signaling axis.

In sum, our consistent findings in the KLB-deficient models and *Fgf21*^Δliver^ mice suggest that FGF21 is mediating GCGR-stimulated weight loss and EE via central KLB receptor complexes.

## Methods

### Animal models.

Mice were single or group housed on a 12-hour light/12-hour dark cycle (lights on from 0600 to 1800 hours) at 22°C and constant humidity with free access to food and water, except as noted. *Klb*-floxed mice were donated by Stephen Kliewer (UT Southwestern Medical Center, Dallas, Texas, USA), and *Synapsin*-Cre mice were obtained from The Jackson Laboratory (strain 003966). All mice were maintained in our facilities on a C57BL/6J background and fed a standard chow (Teklad LM-485, 5.6% fat) or high-fat diet (58.0 kcal% fat; D12331 Research Diets). All animals were euthanized via rapid decapitation with anesthesia (concentrated isoflurane). Mice were sacrificed between Zeitgeber time (ZT) 5 and 7 (where ZT 0 equals time of lights on) for all experiments. Tissues were collected and flash frozen, and plasma was collected from centrifuged (3000*g* for 10 minutes) trunk blood for further analysis.

### ICV and peripheral minipump procedure.

ICV administration of KLB antagonist peptide was conducted as previously described (69). Briefly, 8-week-old male C57BL/6J mice were fed HFD for 24 weeks to induce diet-induced obesity. Mice were anesthetized and administered a single dose of 0.28 mg/kg buprenorphine (Buprenex, Henry Schein Animal Health). A cannula was positioned in the right lateral cerebral ventricle (coordinates: anteroposterior, −0.7 mm to bregma; lateral, –1.2 mm to bregma; dorsoventral, −2.2 mm to the cranial surface), fixed to the skull with cyanocrylate, and connected via a polyethylene catheter to a subcutaneous osmotic minipump (ALZET Osmotic Pumps; Durect Corporation). Osmotic minipumps were implanted subcutaneously in the upper back, delivering vehicle (isotonic saline) or 1153 (0.0171 mg/d, equivalent to 0.3 mg/kg, as shown in Figure 5A) for 14 days. For all experiments, the osmotic minipumps were filled the evening before surgery and primed in a water bath overnight at 37°C.

### Indirect calorimetry.

Mice were single housed in a computer-controlled Comprehensive Laboratory Animal Monitoring System (Columbus Instruments) as previously described (13). Volume of O_2_ consumption and CO_2_ production were measured every 15 minutes to determine RQ and EE. Home cage locomotor activity was determined using a multidimensional infrared light beam system. In all studies with genetic modification, mice were acclimated to their housing conditions for at least 1 week prior to initiation of the experimental protocol. DIO mice with central KLB antagonism went through ICV surgery as described above and were placed in indirect calorimetry units on day 4 of IUB288 treatment for acclimation. Measurements were taken over the next 3 days (days 5–7 IUB288).

### Running wheel behavior.

Mice were housed in individual wheel cages starting at 8 weeks old, and wheel-running activity was recorded and analyzed in 6-minute bins using ClockLab software (Actimetrics) as previously described (70). Mice were maintained in 12-hour light/12-hour dark cycle for 3 weeks before being released into constant darkness (DD). For DD analysis, behavior was analyzed across 10 days after release into DD. Free-running period and amplitude were determined using χ^2^ periodogram analysis with significance set to 0.01.

### Peptides and inhibitors.

GCGR agonist (IUB288) was synthesized as previously described (14). KLB antagonist 1153 was pharmacokinetically optimized from short C-terminal FGF21 and FGF19 peptides that bind to KLB and function as antagonists as previously described (40, 71). Native glucagon and insulin (Humulin R) were obtained from American Peptide Co. and Eli Lilly and Co., respectively.

### Glucose and insulin tolerance tests.

Glucose and insulin tolerance tests were performed in 5- to 6-hour fasted 8- to 10-week-old chow-fed, or 24 week-old DIO, male C57BL/6J mice by i.p. injection of glucose (1.5–2 g/kg, 25% *w/v* d-glucose from MilliporeSigma in 0.9% *w/v* saline) or insulin (0.5–0.75 U/kg in 0.9% *w/v* saline). Blood glucose was determined by Ascensia Contour Glucometer.

### Plasma, tissue, and fecal analyses.

Lipids in plasma and tissue samples from 2-hour fasted mice were determined using Infinity Triglycerides (Thermo Fisher Scientific TR22421) and Infinity Cholesterol (Thermo Fisher Scientific TR13421). Hepatic lipid measurements were conducted following extraction as previously described (39, 72). Briefly, liver (40–80 mg) was homogenized for 2 minutes at 30 Hz (×2) using a TissueLyser II (Qiagen), and lipid was extracted using chloroform/methanol (2:1, *v/v*) and ultrapure water. The organic phase was then separated via centrifugation at 425*g* for 20 minutes at room temperature, dried, and reconstituted in chloroform and vortexed. Values are represented as milligrams of TG per gram of liver. Fecal fat content was measured following isolation of fecal lipid as previously described (73). Briefly, 200 mg of fecal matter was collected from cage bedding during indirect calorimetry and ground to a powder using a mortar and pestle. Lipid was isolated using chloroform/methanol (2:1, *v/v*) and saline. The organic phase was separated via centrifugation at 425*g* for 20 minutes at room temperature, dried, and weighed. The amount of lipid was normalized to starting fecal content and multiplied by 100 to obtain fecal fat percentage.

### Quantitative real-time PCR.

Liver RNA was isolated from 2-hour fasted mice using the RNeasy Lipid Mini-Kit (Qiagen), and cDNA was synthesized by reverse transcription PCR using SuperScriptIII, DNase treatment, and anti-RNase treatment according to the manufacturer’s instructions (Invitrogen). Single gene qPCR was performed as previously described (13), while TaqMan Gene Expression Assay was utilized for identification of cre-mediated *Klb* recombination with the following primer/probe sets: *Klb* (Mm00473122_m1, Applied Biosystems) and *Hprt1* (Mm03024075_m1, Applied Biosystems). Data were normalized to housekeeping gene *Rps18*, as noted using the ΔΔCT calculation. See Supplemental Table 1 for a list of primer sets.

### Immunoblot analyses.

Tissue extracts were prepared in lysis buffer (20 mM Tris at pH 6.8; 3.8 mM DTT, 10% glycerol, 1% SDS, and HALT protease inhibitor cocktail, Thermo Fisher Scientific), rotated for 15 minutes at 4°C, and centrifuged for 10 minutes at 20,817*g* at 4°C. Equivalent protein amounts (20 μg) were separated by 7.5% SDS-PAGE. Resolved fractions were transferred to PVDF membrane (Bio-Rad Laboratories, Inc.), and expression was detected using an antibody against UCP1, 1:1000 (14670, Cell Signaling Technology) normalized to total lane protein by TGX stain-free technology. Vinculin, 1:1000 (4650, Cell Signaling Technology) immunoblot provided as loading control. Immunoblots were labeled with goat anti-rabbit horseradish peroxidase–conjugated secondary antibody, 1:10,000 (7074, Cell Signaling Technology) and protein bands detected and quantified using Clarity ECL, ChemDoc imaging system, and Image Lab 5.0 software (Bio-Rad Laboratories, Inc.).

### Fluorescent immunohistochemistry.

Pancreata were dissected from the 1153-treated cohort and fixed overnight in 4% formaldehyde diluted in 1× PBS at 4°C, then embedded in paraffin or OCT (Tissue-Tek 4583) as previously described (74). Sections were cut to 6 μm and then blocked using 5% normal donkey serum in 1% bovine serum albumin in 1× PBS for 1 hour at room temperature. Sections were then incubated with primary antibodies overnight at 4°C: guinea pig α-insulin, 1:1000 (0564, Dako); mouse α-glucagon, 1:4000 (G2654, MilliporeSigma); and goat somatostatin, 1:1000 (7819, Santa Cruz Biotechnology). Cy-2–, Cy-3–, and Cy-5–conjugated α-goat, α–guinea pig, or α-mouse IgG secondary antibodies 1:500 (Jackson ImmunoResearch Laboratories) were used to detect indirect immunofluorescence. Slides were imaged using a Zeiss LSM710 confocal microscope, and the images were processed by Zen software (Zeiss).

### Statistics.

All data are represented as mean and SEM. Statistical significance was determined using unpaired 2-tailed Student’s *t* tests or, where appropriate, analysis of covariance, 2-way ANOVA with multiple comparisons Sidak posttest, or 3-way ANOVA. Statistics were completed using GraphPad Prism version 6.0 for Macintosh (GraphPad Software) and significance assigned when *P* < 0.05.

### Study approval.

All studies were approved by and performed according to the guidelines of the Institutional Animal Care and Use Committee of the University of Alabama at Birmingham.

## Author contributions

SRN and KMH were responsible for study conception and design, data analyses and interpretation, and drafting the article; SRN, JA, NP, SEC, THP, TK, and CH generated experimental data; JRP and KLG analyzed circadian locomotor data; MEY, KLG, BF, RD, CSH, and AK advised on study concept and critical revision of the article. KMH is the guarantor of this work and, as such, had full access to all the data in the study and takes responsibility for the integrity of the data and the accuracy of the data analysis.

## Supplementary Material

Supplemental data

## Version 1 01/07/2021

In-Press Preview

## Version 2 02/22/2021

Electronic publication
