# Supplementary material for: Glucagon receptor signaling regulates weight loss via central KLB receptor complexes
Source: JCI Insight. 2021 Feb 22;6(4):e141323. doi: 10.1172/jci.insight.141323 (PMC7934938; doi:10.1172/jci.insight.141323)
Supplement: Supplemental data [file jciinsight-6-141323-s030.pdf]

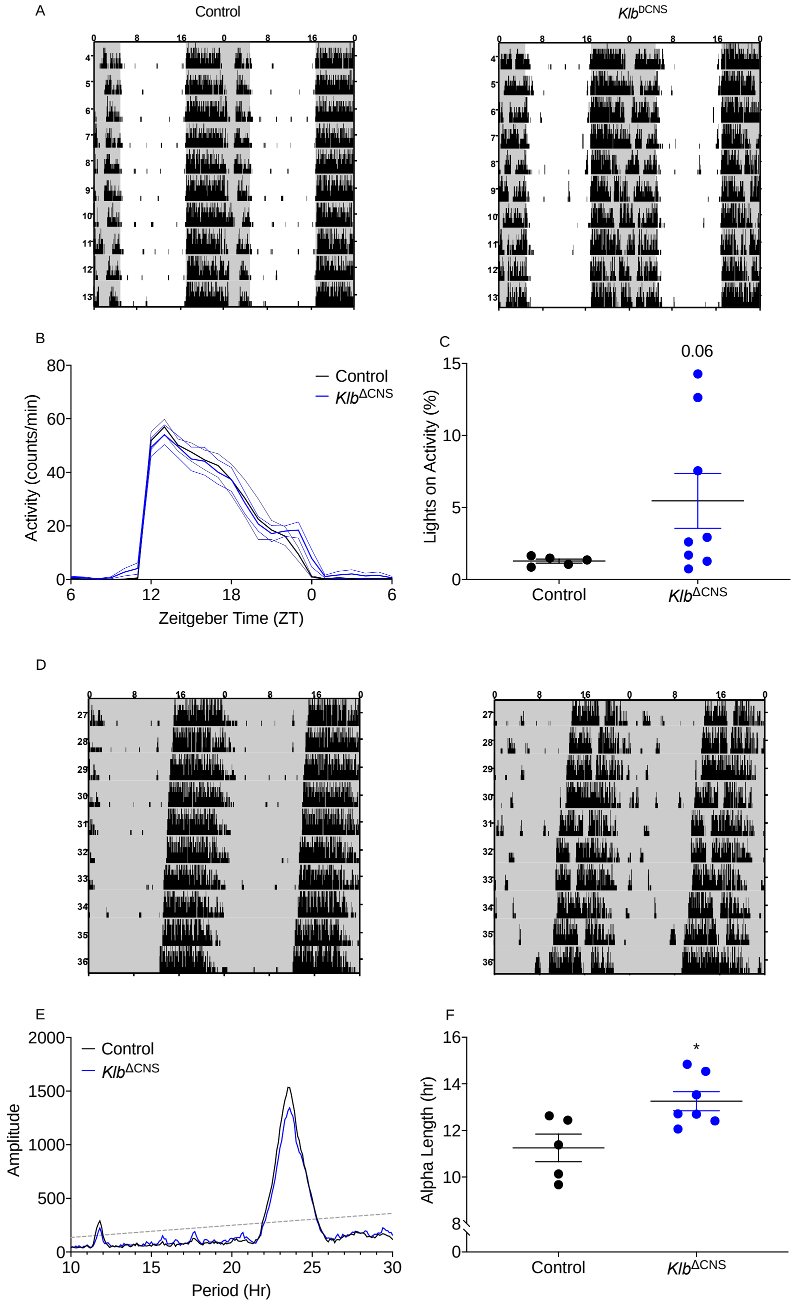

Supplementary Figure 1. Circadian Locomotor Behavior in *Klb*$^{\Delta CNS}$ mice

Representative light-dark double plotted actograms of wheel-running activity for control (left) and *Klb*$^{\Delta CNS}$ mice (right) (A). Average wave plots summarizing wheel-running light-dark activity (B) and % lights on activity (C) (n=5-8). Representative free-running double plotted actograms in constant dark (D). Representative χ^2 periodogram (E) and average alpha length (F; n=5-7) in control and *Klb*$^{\Delta CNS}$. All data are represented as mean ± SEM. *p<0.05, Student's t-test

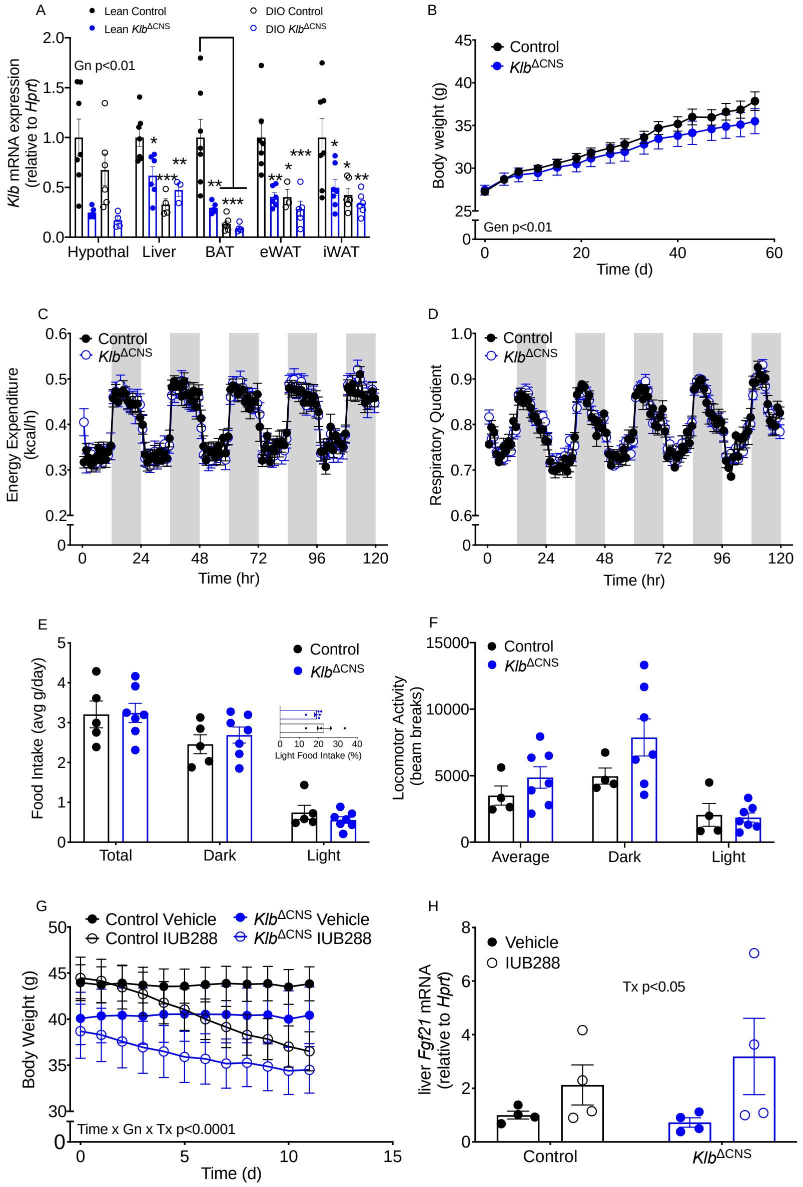

Supplementary Figure 2. Diet-induced obesity and GCGR agonism in *Klb*$^{\Delta CNS}$ mice

Klb expression in lean, chow-fed mice (8wk-old n=5-7) and DIO, HFD-fed mice (16wk-old mice n=4-6) (A). Absolute body weight (B) in control and *Klb*$^{\Delta CNS}$ fed HFD for 8wk (n=10-14). Diurnal EE (C), respiratory quotient (D), food intake (E) and % light food intake (E inset), and locomotor activity (F) in control and *Klb*$^{\Delta CNS}$ for remaining 7d of HFD after 3d chow (n=5-7). Absolute body weight (G) in control and *Klb*$^{\Delta CNS}$ mice treated with IUB288 for 12d. Liver Fgf21 expression (H) after 12d IUB288 in control and *Klb*$^{\Delta CNS}$ mice. All data are represented as mean ± SEM, *p<0.05, **p<0.01, ***p<0.001 compared to lean vehicle controls, two-way ANOVA. Panel A: Main effect of genotype (p<0.01). Panel B: Main effect of genotype in hypothalamus (p<0.01). Panel G: Main effect of treatment (p<0.05). GCGR Agonist: IUB288

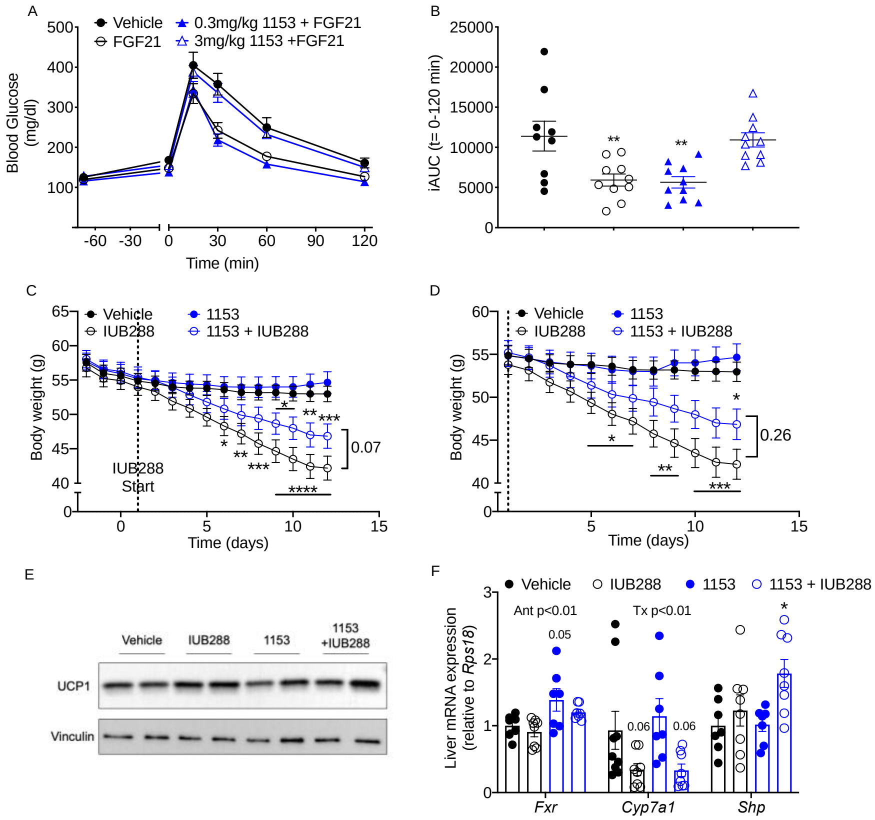

Supplementary Figure 3. GCGR-agonism in mice with KLB Antagonism

Glucose tolerance test (A, 5h fast) and iAUC (B) in 10wk-old lean mice (n=10). 1153 (0.3mg/kg or 3mg/kg) or vehicle administered at t=-70m and FGF21 or vehicle administered at t=-60m, before glucose (2g/kg) administered at t=0m. Absolute body weight in control and 1153 DIO mice from ICV surgery start (C; t=-2d) and from IUB288 start (D) (n=12-14). Dotted line indicates start of IUB288 (t=1d). Representative BAT UCP1 from 6 individual samples (E) and gene expression of Liver *Fxr* and downstream *Fxr*-targets (F; n=7-9). *p<0.05, **p<0.01, ***p<0.001, ****p<0.0001 compared to respective genotypic controls. $^{\#}$p<0.05, $^{\#\#}$p<0.01, $^{\#\#\#\#}$p<0.0001 between IUB288 and 1153 + IUB288 groups, two-way ANOVA. Panel E: main effect of 1153 (p<0.01) on *Fxr* and main effect of treatment on *Cyp7a1* (p<0.01). GCGR Agonist: IUB288. KLB Antagonist: 1153.

Supplementary Table 1. qPCR primers

**Gene**	**Forward (5'-3')**	**Reverse (5'-3')**
Abca1	CGT TTC CGG GAA GTG TCC TA	GCT AGA GAT GAC AAG GAG GAT GGA
Acaca	CTT CCT GAC AAA CGA GTC TGG	CTG CCG AAA CAT CTC TGG GA
Hmgcr	GTG TTC AAG GAG CAT GCA AAG	AGC CAT CAC AGT GCC ACA TAC
Srebp-1	GAG GAC CTT TGT CAT TGG CTG	TAC AGA GCA AGA GGG TGC CAT
Fxr	CAC AGC GAT CGT CAT CCT CTC T	TCT CAG GCT GGT ACA TCT TGC A
Cyp7a1	GGG ATT GCT GTG GTA GTG AGC	GGT ATG GAA TCA ACC CGT TGT C
Shp	CAT GGC CTC TAC CCT CAA GAA C	GTC ACC TCA GCA AAA GCA TGT C
Rps18	TTC TGG CCA ACG GTC TAG ACA AC	CCA GTG GTC TTG GTG TGC TGA
